# Doctor Making

**Published:** 1876-01

**Authors:** 


The Bistoury.

ELMIRA, N. Y., JAN, 1876.

The BISTOURY is published Quarterly, upon the 1st of April, July, October and January, at Fifty Cents a year, in advance.

### DOCTOR-MAKING.

One is astonished, upon visiting any of the Medical Colleges of our cities, to see the number of *boys* there assembled, striving to obtain sufficient information to enable them to gain a livelihood, without manual labor. To call these young people students, is a burlesque upon the term. Many of them have not been prepared with a common school course of study, much less advanced to the dignity of a classical education, which, alone, at this day, can prepare a man thoroughly, to enter upon the study of medicine as a profession. No examination as to qualification for the pursuit of such a study, is required in any of these so-called colleges,—save the honorable exception of Harvard. A chin-whisker, and the fee in cash, for the Professor's tickets, and thirty dollars for the parchment, is the sure passport to these institutions, and floods our country yearly, with about three thousand of the worst specimens of "doctors" that can be found anywhere in the civilized world. How under the sun these professors can find it in their consciences to recommend these youths for diplomas, as doctors of medicine, is beyond our ability to fathom. Of course, among them are a few young *men* of sufficient culture and attainment to warrant the belief that they will rise to eminence and usefulness in their profession; but these school-boys, who have "read medicine" with a preceptor, (i. e., held the horses while the "old doctor" visited his patients,) for a year or two, and this preparation constituting their only qualification, why, it is an imposition upon the people, to graduate them. It is utterly impossible for them to become physicians;—they lack education, application, and honesty of purpose. Their sole intent is to make money, and that, as easily as possible. The moment they obtain their diplomas, study ceases, and an attempt at money-making begins. Books, if they have any, are discarded, medical journals never thought of, and the youngster settles down to await the coming of his first patient. When a few of the superfluous doctors in the neighborhood die, or are driven away by famine, our young sprig may have a call or two, that may eventually grow into a satisfactory "practice." And this will be the history of nine-tenths of them that do not abandon the "profession," and find some useful employment, before the end of the first year. The country is over-crowded by just this sort of doctors—you will find them everywhere—"there's millions of 'em," seemingly, and the community want no more of them.

Fathers and mothers! For heaven's sake, send your "smart boys" elsewhere than to a medical college! A lunatic asylum would be preferable—perhaps for both of you. There is a growing demand for educated and skilled mechanics, builders, and engineers, but no room for third-rate doctors. It is *cheaper*, no doubt, to educate (?) your boy for a doctor—the more's the pity, but it is more honorable to make a useful, skillful man of him.

